# Impact of main residential locations on depressive symptoms among older adults in China: A Blinder–Oaxaca decomposition analysis

**DOI:** 10.3389/fpubh.2022.934940

**Published:** 2022-12-05

**Authors:** Yuqing Cheng, Qiutong Yu, Wei Li, Genyong Zuo

**Affiliations:** ^1^Center for Health Management and Policy Research, School of Public Health, Cheeloo College of Medicine Shandong University, Jinan, Shandong, China; ^2^NHC Key Laboratory of Health Economics and Policy Research, Shandong University, Jinan, Shandong, China

**Keywords:** depressive symptoms, older adults, Blinder-Oaxaca decomposition, rural-urban migrants, urbanization

## Abstract

**Background:**

With the development of urbanization in China, the scale of internal migration and the number of immigrants among older adults are increasing. This requires paying attention to the living conditions and environment of immigrants. Many studies note a gap in the prevalence of depressive symptoms among older adults living in different main residential locations. However, few studies have examined the extent to which main residential locations influence depressive symptoms among older adults. This study aims to quantify the effect of main residential locations on depressive symptoms.

**Methods:**

For this study, we used data from the 2018 Chinese Longitudinal Health and Longevity Survey and randomly selected 8,210 individuals aged 65 years and older were from the community to determine the effect of main residential locations on depressive symptoms among older adults. We further used the Blinder–Oaxaca decomposition method to quantify the explanatory factors of depressive symptom gaps among older adults and to estimate the relative effect of individual characteristics on depressive symptoms.

**Results:**

In this study, we noted significant differences in depressive symptoms among older adults in different main residential locations. Rural–urban migrants had higher depressive symptom scores (7.164). According to the Blinder–Oaxaca decomposition analysis, the high proportion of the depressive symptom gap can be explained by years of education, income, and exercise among different main residential locations groups. In addition, in the main parts of the explained differences, the proportions of the limitation of activities of daily living (2.28, 0.46, and −52.11%) showed opposite effects, while their share in different main residential locations groups varied widely.

**Conclusion:**

Urbanization has resulted in more rural people moving to urban areas in China; Rural–urban migrants have the highest prevalence of depressive symptoms, which needs attention. Thus, there is an urgent need to integrate the health insurance and pension policy for urban and rural residents. This study provides a basis for formulating health policies and promoting the mental health of older adults in China as well as in low- and middle-income countries.

## Introduction

Urbanization and population aging have become the main trends worldwide, especially in China. Following the acceleration of urbanization, a large number of rural people work or have settled in urban areas. Compared with the data collected during the sixth population census of China in 2010, the internal migration population increased by 69.73% during the seventh population census conducted by the National Bureau of Statistics in 2020 (1). The mental health status of older adults in the internal migration population requires more attention as they are a vulnerable group. The decline in the health status, stress-coping ability, and social networks of older adults may affect their mental health.

Depressive symptoms are common among people with chronic diseases, cognitive impairment, or disabilities, especially older adults. According to the World Health Organization (WHO), ~280 million people worldwide suffer from depressive symptoms, 5.7% of whom are adults older than 60 years (2). Although there are effective treatments for mental disorders, more than 75% of the patients in low- and middle-income countries do not have access to treatment (3). The WHO estimated that three-quarters of this burden occurred in low- and middle-income countries (4). A meta-analysis study has shown that the overall prevalence of depressive symptoms has reached 23.6% among older adults in China (5); this is significantly higher than that reported more than 20 years ago (6). Simultaneously, the development of urbanization has caused a disparity in mental health between people in urban and rural areas. Studies in China have consistently reported that the prevalence of depressive symptoms among older adults in rural areas is higher than among those in urban older adults (7–11).

To the best of our knowledge, when studying the location of residence, urbanization is, in general, used as the basis for grouping. The previous literature has documented the relationship between urbanization and depressive symptoms in China (12), the potential influencing factors of urbanization on depressive symptoms, and the role of mediating variables on depressive symptoms among rural–urban migrants, such as neighborhood social capital (13). In addition, although individual and urban–rural differences have been examined in the literature (11), it does not accurately quantify urban–rural differences and the extent to which the characteristics of each individual can explain the gap resulting in depressive symptoms. Therefore, there is a need for appropriate statistical methods to evaluate the degree to which relevant individual characteristics explain the difference between depressive symptoms among people in urban and rural areas. The Blinder–Oaxaca decomposition method has been used to examine the wage gap between men and women in the United States to further assess the average of various factors in the variables as well as the effect of wage differences caused by non-explanatory variables (14). Since then, it has been applied to health research many times (15, 16). Therefore, we use the Blinder–Oaxaca decomposition to quantify the average differences between groups by decomposing variables into explainable differences and unexplained differences caused by unobserved differences between groups (17).

The study aims to assess whether there is a gap in the prevalence of depressive symptoms among older adults in main residential locations and, in the case of a gap, quantitatively estimate the influence of main residential location on depressive symptoms among older adults. We expected that by focusing on the prevalence of depressive symptoms among older domestic immigrants, we could explore the effect of the main residential location on effectively narrowing the mental health gap between urban and rural older adults. This is conducive to reducing the prevalence of depressive symptoms and promoting the mental health of older adults.

### Conceptual framework and hypotheses

The acculturation theory can be used to explain the relationship between main residential locations and depressive symptoms among older adults. The acculturation theory states that immigrants may experience the pressure of cultural adaptation on interacting with local residents, which can result in physical and psychological problems (18). Previous studies have shown that when people stay longer in a place, they tend to adapt to the culture of the place. As a result, their health improves (19–21). In general, the duration of residence in a settlement relatively affects the health status of immigrants by changing their education, income, and health awareness. Based on this, we propose a hypothesis regarding the relationship between the main residential location and depressive symptoms.

Hypothesis: Rural–urban migrants have higher depressive symptom scores than permanent urban and rural residents.

## Methods

### Study population

**Figure 1:**
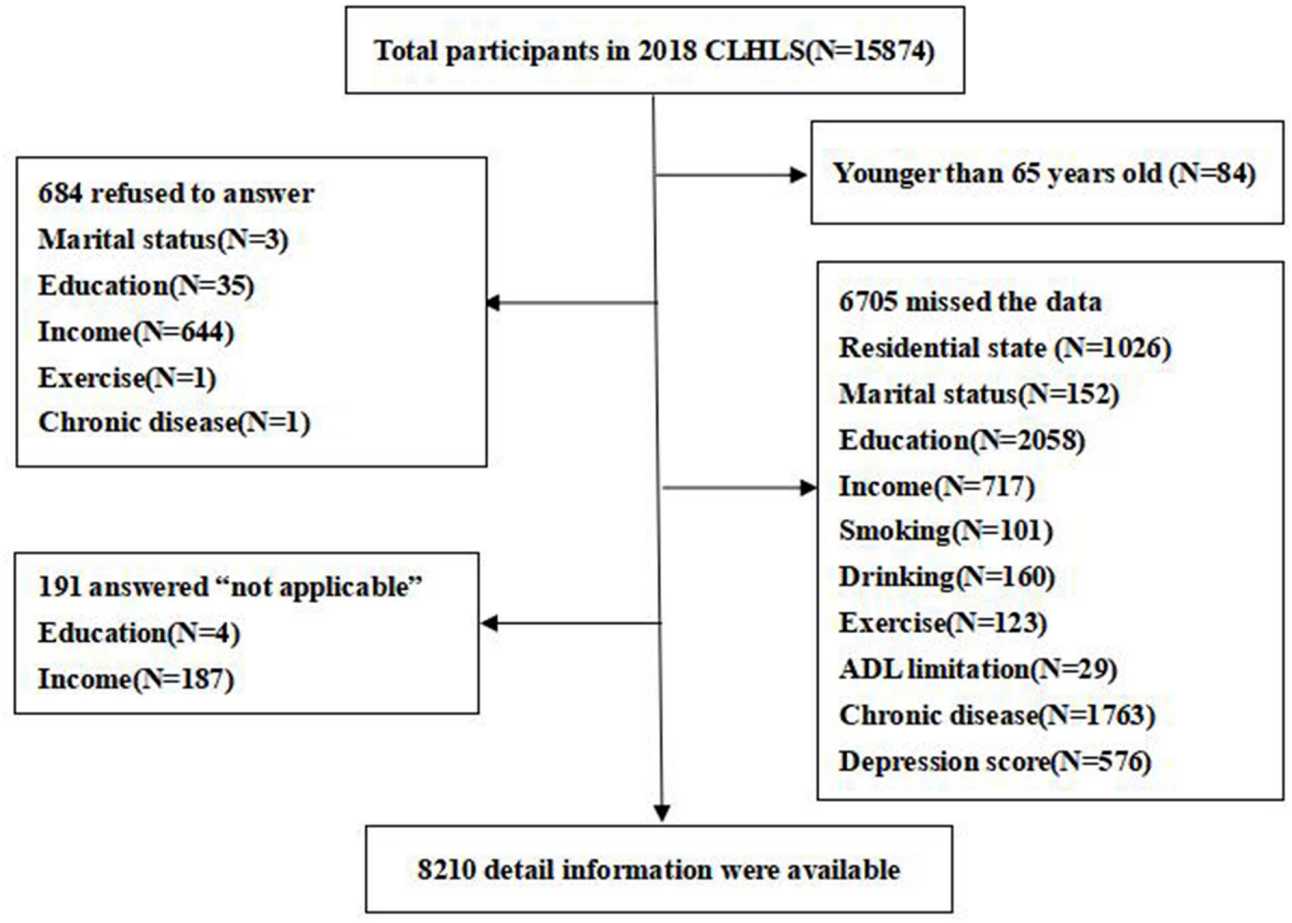
Study flowchart of participants selection (aged 65 years or older) from the Chinese Longitudinal Healthy Longevity Survey 2018 survey data.

We obtained data from the 2018 Chinese Longitudinal Healthy Longevity Survey (CLHLS), which was organized by the Health Aging and Development Research Center of Peking University/National Institute of Development. The project launched a baseline survey in 1998, which has been conducted eight times so far, covering the family structure, living arrangements, social participation, and quality of life of adults aged 65 and older. The survey involved face-to-face interviews with interviewees in the form of household visits to the community to collect information in Chinese. Investigators were selected by recruiting volunteers who required people with a bachelor's degree or higher to apply. Through the unified pre-job training, familiarization with the items of the questionnaire, question and answer skills, and the instructor led them to conduct interviews. The interviews were free of questions and answers. The cumulative number of visitors exceeded 110,000, of whom 67.4% were ≥80 years old. The sampling design of the survey adopted the multi-stage and unequal-proportion target random sampling method. First, ~50% of the counties, county-level cities, or districts in 23 provinces, municipalities, or autonomous regions were randomly selected. In these areas, all centenarians who were alive and voluntarily participated in the survey were interviewed at home. Age and sex were randomly selected according to the number of centenarians in order to ensure that the number of respondents in other age groups is roughly similar to that of centenarians, and that the sex ratio of the sample is roughly similar. In 2018, we focused on all the older adults. The 2018 CLHLS data included 15,874 observers aged ≥65 years. Those who were younger than 65 years (*n* = 84), had missed data (*n* = 6,705), were not applicable (*n* = 191), and refused to participate (*n* = 684) were excluded. The remaining 8,210 older adults were included in the study (Figure 1). To maintain a nationally representative sample, the weight of age, sex, and residence in the 2018 CLHLS sample was based on categories of respondents with similar sociodemographic characteristics (22, 23); there were 7,042 weighted samples.

### Measures of depressive symptom score

The depressive symptom score, which was a continuous variable, was selected as the dependent variable. The 2018 CLHLS questionnaire evaluated depressive symptom scores among older adults using the Center for Epidemiologic Studies Short Depression Scale (CES-D). The CES-D scale was developed by Radloff at the National Institute of Mental Health in 1977 and is widely used in epidemiological surveys to screen for depressive symptoms. The scale included three positive questions and seven reverse questions. The answers are “always” = “3,” “often” = “2,” “sometimes” = “1,” and “rarely” or “never” = “0.” After reversing the three positive impact items, a total score of 10 items was summed, with “depressive symptoms” assigned a value of “1” for those with a total score of ≥10; otherwise, it was normal, which was assigned a value of “0” (24, 25). The higher the depressive symptom score, the more severe the depressive symptoms. The purpose of determining whether there are depressive symptoms or not is to describe the prevalence rate of depressive symptoms among older adults in different groups and to further intuitively analyse the gap in depressive symptoms in different main residential locations. Related studies have proven that the CES-D scale can be used in Chinese and reported good reliability and validity (26). In this study, Cronbach's alpha coefficient of the CES-D scale was 0.774.

### Classification of main residential locations

The existing literature proposes many methods for classifying main residential locations. For example, residents were previously classified according to the criteria of living in the local area for 6 months within a year or 5 or more years (27, 28). However, China's hukou system has affected the distribution of basic public services. This system divides hukou into urban hukou and rural hukou according to blood inheritance and geographical location. Under this system, people's employment and social welfare are related to household registration. In general, people with urban hukou enjoy far more benefits than rural hukou, such as education, healthcare, and social security (29, 30). Currently, in China, compared with older adults with rural hukou who can only enjoy basic pension benefits, those with urban hukou are much better off after retirement. However, the vast majority of people who migrate from rural to urban areas are unable to obtain urban hukou due to some reasons, such as a low level of education, so they are still associated with their place of birth and cannot enjoy the treatment guarantee of the urban population. With the rapid development of urbanization in China, more and more people are migrating from rural areas to urban areas, forming a subgroup. We take account of the place of residence, and hukou and residence time have a greater impact on older adults, especially mental health. Therefore, this study used a measure that combines the interviewees' residence and hukou identity information and the number of years of residence. Based on these, three types of main residential locations were determined: (a) urban residents, meaning that the respondents had an urban hukou and had lived in the city for 10 years or more; (b) rural–urban migrants, indicating that interviewees had a rural hukou, but live in urban areas; and (c) rural residents, implying that respondents have a rural hukou and have lived in rural areas for 10 years or more (31, 32).

### Covariates

Population baseline characteristics included (a) sociodemographic factors: age (continuous variable), sex (male or female), and marital status (married/cohabiting or single); (b) socioeconomic status (SES): years of education (continuous variable) and income (continuous variable); (c) lifestyle: smoking (yes or no), drinking (yes or no), and exercise (yes or no); and (d) health status: activities of daily living limitation (ADL limitation; yes or no) and chronic diseases (yes or no). Among them, the assignment of chronic diseases was based on whether the patient had been diagnosed with a certain disease, which we defined as suffering from chronic disease if interviewers had one or more types of chronic disease. We collected data on chronic diseases in the form of self-reports by interviewees. Table 1 shows covariate collection and assignment.

**Table 1:** Covariates and assignment.

**Variable**	**Question**	**Assignment**
**Sociodemographic characteristics**		
Age	Validated age	
Gender	Sex	1. Male (1) 2. Female (0)
Married/cohabiting	Current marital status	1. Married and living with spouse (1 = married) 2. Married but not living with spouse (1 = married) 3. Divorced (0 = single) 4. Widowed (0 = single) 5. Never married (0 = single)
**SES**
Education (years)	How many years did you attend school?	Continuous variables
Income	What was the income per capita of your household last year?	Continuous variables
**Life styles**		
Smoking	Do you smoke at the present time?	1. Yes (1) 2. No (0)
Drinking	Do you drink alcohol at the present time?	1. Yes (1) 2. No (0)
Exercise	Do you do exercises regularly at present?	1. Yes (1) 2. No (0)
**Health status**		
ADL limitation	For at least the last 6 months have you been limited in activities people usually do, because of a health problem?	1. Yes, strongly limited (1 = yes) 2. Yes, limited (1 = yes) 3. Not limited (0 = no)
Chronic disease	Are you suffering from any of the following chronic disease? (Diagnosed by hospital?)	1. Yes (1) 2. No (0)

ADL limitation, activities of daily living limitation. Chronic diseases include hypertension, diabetes, heart disease, stroke, cerebrovascular disease, bronchitis, emphysema, asthma, pneumonia, pulmonary tuberculosis, cataracts, glaucoma, cancer, prostate tumor, gastric or duodenal ulcer, Parkinson's disease, bedsores, arthritis, dementia, epilepsy, cholecystitis, cholelithiasis disease, blood disease, rheumatism or rheumatoid disease, chronic nephritis, galactophore disease, uterine tumor, hyperplasia of prostate, hepatitis, and others.

### Statistical analysis

We used STATA/MP 15.0 (Stata Corp, College Station, TX, USA) for all the analyses. The analysis process in this study was divided into three steps. First, descriptive statistics were expressed as mean and standard deviations (SD) or numbers and percentages. Second, ordinary least squares regression was used to estimate whether there was a significant correlation between the main residential location and depressive symptoms. In the regression analysis, we divided the participants into three models according to the sociodemographic characteristics and behavioral health: model 1 (only considering main residential location and depressive symptom score), model 2 (considering the sociodemographic characteristics and socioeconomic status), and model 3 (considering both the sociodemographic characteristics and lifestyle and health status). The Blinder–Oaxaca decomposition method was then used, which acts as the average difference of results between different groups. The depressive symptom gap between the two groups was divided into two parts, namely, the “explained” group difference and the difference of other factors that cannot be explained (14, 15, 33). The model is represented as follows:


$${\bar{y}}_{a}-{\bar{y}}_{b}=\left({\bar{x}}_{a}-{\bar{x}}_{b}\right)\;\beta_{b}+{\bar{x}}_{b}\;\left(\beta_{a}-\beta_{b}\right),$$

where ***a*** and ***b*** represent the groups of older adults with different main residential locations, $({\bar{x}}_{a}-{\bar{x}}_{b})\beta_{b}$ is referred to as the “explained component”, and ${\bar{x}}_{b}(\beta_{a}-\beta_{b})$ is the “unexplained component.” By calculating the average difference in depressive symptom scores among groups, the explainable factors resulting in the depressive symptom gap between groups were estimated and quantified (34). According to the principle of Blinder–Oaxaca decomposition, we acquired three models and performed a comparative analysis of urban residents, rural–urban migrants, and rural residents.

## Results

### Participants' characteristics

Table 2 presents the descriptive characteristics of the study participants. A total of 7,042 (weighted) older adults participated in this study. Of these, 1,926 (27.35%) were urban residents, 1,886 (26.78%) were rural–urban migrants, and 3230 (45.87%) were rural residents. Of all the participants, 23.02% exhibited depressive symptoms. Rural–urban migrants had the highest depressive symptom score (7.164), followed by rural residents; urban residents had the lowest depressive symptom scores. This finding was consistent with our hypothesis. In general, compared with other older adults, the majority of those who had depressive symptoms were woman, single persons, people who liked smoking and drinking alcohol, could not perform their daily activities, and were temporary city residents.

**Table 2:** Descriptive characteristics of study participants from CLHLS 2018 (weighted).

	**Total**	**Urban resident**	**Rural–urban migrant**	**Rural resident**
Total	7,042 (100.00%)	1,926 (27.35%)	1,886 (26.78%)	3,230 (45.87%)
Depressive symptoms	1,621 (23.02%)	414 (21.50%)	497 (26.35%)	710 (21.98%)
CES-D score^a^	6.867 (4.351)	6.761 (4.706)	7.164 (4.422)	6.758 (4.073)
**Sociodemographic characteristics**
Age^a^	72.391 (6.592)	72.935 (6.668)	72.494 (6.783)	72.006 (6.407)
Male	3,330 (47.29%)	917 (47.61%)	908 (48.14%)	1,505 (46.59%)
Married/cohabiting	5,167 (73.37%)	1,453 (75.44%)	1,337 (70.89%)	2,377 (73.59%)
**SES**
Education (years)^a^	4.905 (4.310)	8.268 (4.643)	3.671 (3.469)	3.620 (3.366)
Income (yuan)^a^	43,534.45 (36,014.88)	70,883.57 (30,640.93)	33,993.25 (32,731.05)	32,799.24 (32,072.14)
**Life styles**
Smoking	1,336 (18.97%)	283 (14.69%)	402 (21.31%)	651 (20.15%)
Drinking	1,321 (18.76%)	310 (16.10 %)	378 (20.04 %)	633 (19.60%)
Exercise	2,928 (41.58 %)	1,175 (61.01%)	730 (38.71%)	1,023 (31.67%)
**Health status**
ADL limitation	1,462 (20.76%)	386 (20.04%)	417 (22.11%)	659 (20.40%)
Chronic disease	5,943 (84.39%)	1,729 (89.77%)	1,563 (82.87%)	2,651 (82.07%)

a Continuous variables are reported as mean values and standard deviations; categorical variables are reported as numbers and percentages.

CES-D, Center for Epidemiologic Studies Short Depression Scale; ADL limitation, activities of daily living limitation.

### Ordinary least square regression analysis

Table 3 presents the results of the ordinary least square regression analysis. In the unadjusted model, rural–urban migrants were at higher risk of exhibiting depressive symptoms. Model 1 considered sociodemographic characteristics and socioeconomic status. With the urban residents as the reference group, the coefficient of rural–urban migrants was −0.320, and the coefficient of rural residents was −0.719; both passed the 5% statistical test, indicating that the main residential location negatively affected depression scores. Model 2 not only considered the sociodemographic characteristics and socioeconomic status but also adjusted for lifestyles and health status. The results showed that the relationship between rural–urban migrants and depressive symptoms was significantly stronger.

**Table 3:** Ordinary least squares regression for main residential location and total depressive symptom score (weighted).

**Model specifications**	**All sample**		
	**Unadjusted model**	**Adjusted model 1**	**Adjusted model 2**
Urban resident	(Ref.)		
Rural–urban migrant	0.403^**^ (0.126,0.679)	−0.320^*^ (−0.638, −0.002)	−0.447^**^ (−0.759, −0.136)
Rural resident	−0.003 (−0.249,0.242)	−0.719^***^ (−1.015, −0.424)	−0.924^***^ (−1.215, −0.633)
**Sociodemographic characteristics**
Age		0.024^**^ (0.008, 0.041)	0.001 (−0.015, 0.018)
Male		−0.543^***^ (−0.760, −0.327)	−0.399^***^ (−0.635, −0.163)
Married/cohabiting		−0.836^***^ (−1.085, −0.586)	−0.876^***^ (−1.119, −0.633)
**SES**
Education		−0.031^*^ (−0.060, −0.001)	−0.004 (−0.033, 0.024)
Income		−0.000015^***^ (−0.000018, −0.000012)	−0.000013^***^ (−0.000016, −0.000010)
**Life styles**
Smoking			0.186 (−0.095, 0.467)
Drinking			−0.643^***^ (−0.919, −0.368)
Exercise			−1.426^***^ (−1.632, −1.220)
**Health status**
ADL limitation			1.537^***^ (1.291, 1.783)
Chronic disease			0.295^*^ (0.023, 0.566)

95% confidence intervals (CI) in parentheses.

ADL limitation, activities of daily living limitation.

* *p* < 0.05.

** *p* < 0.01.

*** *p* < 0.001.

### Decomposition of the depressive symptom gap between urban residents and rural–urban migrants (weighted)

As shown in Table 4 (model 1), the depressive symptom score of urban residents was 0.403 lower than that of rural–urban migrants (*p* < 0.05). The gap in depressive symptoms is mainly explained by individual attribute differences (499.75%), indicating that the explained component “overexplains” the difference in the average score between the two groups. Assuming that this value is 100%, the difference in the explained component simply explains all the differences in depressive symptom scores. However, if it is >100%, it signifies that once the explained component is controlled, the score gap is reversed. The depressive symptom score of rural–urban migrants was not higher than that of urban residents but was lower than that of urban residents. This is mainly because of the difference in depressive symptoms according to income (49.90%). In addition, the difference in education years (38.53%) was also a significant reason for the difference in depressive symptom scores between the two groups. By contrast, the differences in scores caused by other explanatory factors were significantly less.

**Table 4:** Blinder–Oaxaca decomposition of the gap in depressive symptoms between the urban resident and rural–urban migrant samples (weighted).

	**1. Urban resident (1) and Rural**–**urban migrant (2)**	
	**Mean (95%CI)**	**N/%**
Group_1 (urban resident)	6.761^***^ (6.563, 6.959)	1,926
Group_2 (rural–urban migrant)	7.164^***^ (6.972, 7.355)	1,886
Difference	−0.403^**^ (−0.678, −0.127)	100.00%
Explained difference	−2.014^***^ (−2.355, −1.673)	499.75%
Unexplained difference	1.611^***^ (1.189, 2.033)	−399.75%
**Explanatory variables**
**Sociodemographic factors**
Age	−0.010 (−0.026, 0.006)	0.50%
Male	0.005 (−0.023, 0.033)	−0.25%
Married/cohabiting	−0.015 (−0.036, 0.007)	0.74%
**SES**
Education (years)	−0.776^***^ (−1.055, −0.496)	38.53%
Income	−01.005^***^ (−1.214, −0.797)	49.90%
**Life styles**
Smoking	−0.004 (−0.037, 0.028)	0.20%
Drinking	−0.012 (−0.033, 0.008)	0.60%
Exercise	−0.174^***^ (−0.260, −0.088)	8.64%
**Health status**
ADL limitation	−0.046 (−0.101, 0.010)	2.28%
Chronic disease	0.023 (−0.010, 0.057)	−1.14%

95% confidence intervals (CI) in parentheses.

N, number; ADL limitation, activities of daily living limitation.

** *p* < 0.01.

*** *p* < 0.001.

Combined with descriptive statistics, in addition to the unexplained differences that result in the gap in depressive symptoms, other attributes of urban residents, such as better marital status and more opportunities for exercise, make the depressive symptom scores lower than the rural–urban migrants. The negative explained difference was counteracted by a larger positive unexplained difference. Rural–urban migrants reported more severe depressive symptoms for a given set of characteristics.

### Decomposition of the depressive symptom gap between urban residents and rural residents (weighted)

In model 2 of Table 5, the depressive symptom score of rural residents was 6.758 points, which was 0.003 points lower than that of urban residents. It is worth noting that the proportion of the explained difference in the Blinder–Oaxaca decomposition of depressive symptom scores was −43,100%. This suggests that the explained difference plays an inverse role in driving a gap in the depressive symptom scores between the two groups. This means that after considering these covariates, the score gap between the two groups will not increase but will decrease further. Years of education (48.72%), income (32.8%), and exercise (14.77%) were the most important factors that affected depressive symptoms.

**Table 5:** Blinder-Oaxaca decomposition of the gap in depressive symptoms between urban resident and rural resident samples (weighted).

	**2. Urban resident (1) and Rural resident (2)**	
	**Mean (95%CI)**	**N/%**
Group_1 (urban resident)	6.761^***^ (6.563, 6.959)	1,926
Group_2 (rural resident)	6.758^***^ (6.607, 6.908)	3,230
Difference	0.003 (−0.246, 0.252)	100.00%
Explained difference	−1.293^***^ (−1.585, −1.002)	−43,100.00%
Unexplained difference	1.297^***^ (0.924, 1.669)	43,233.33%
**Explanatory variables**
**Sociodemographic factors**
Age	0.019 (−0.005, 0.043)	−1.47%
Male	0.002 (−0.004, 0.008)	−0.15%
Married/cohabiting	−0.018 (−0.043, 0.007)	1.39%
**SES**
Education (years)	−0.630^***^ (−0.854, −0.407)	48.72%
Income	−0.480^***^ (−0.652, −0.308)	37.12%
**Life styles**
Smoking	−0.023 (−0.046, 0.001)	1.78%
Drinking	0.042^**^ (0.013, 0.072)	−3.25%
Exercise	−0.191^***^ (−0.283, −0.098)	14.77%
**Health status**
ADL limitation	−0.006 (−0.042, 0.031)	0.46%
Chronic disease	−0.008 (−0.037, 0.021)	0.62%

95% confidence intervals (CI) in parentheses.

N, number; ADL limitation, activities of daily living limitation.

** *p* < 0.01.

*** *p* < 0.001.

### Decomposition of the depressive symptom gap between rural residents and rural–urban migrants (weighted)

In model 3 of Table 6, the depressive symptom score of rural residents was 6.758 points, which was 0.406 points lower than that of rural–urban migrants (*p* < 0.001). Among the explainable differences in depressive symptoms, exercise (77.46%) and income (46.48%) were more likely to be explained. Interestingly, the proportion of ADL limitation was positive in the previous inter-group comparison, whereas it was negative in the rural residential and rural–urban migrant groups. Moreover, the proportion was very high.

**Table 6:** Blinder–Oaxaca decomposition of the gap in depressive symptoms between the rural resident and rural–urban migrant samples (weighted).

	**3. Rural resident (1) and Rural**–**urban migrant (2)**	
	**Mean (95%CI)**	**N/%**
Group_1 (rural resident)	6.758^***^ (6.607, 6.908)	3,230
Group_2 (rural–urban migrant)	7.164^***^ (6.972, 7.355)	1,886
Difference	−0.406^***^ (−0.650, −0.162)	100.00%
Explained difference	0.071 (−0.032, 0.175)	−17.49%
Unexplained difference	−0.477^***^ (−0.709, −0.246)	117.49%
**Explanatory variables**
**Sociodemographic factors**
Age	0.011 (−0.005, 0.028)	15.49%
Male	0.014 (−0.013, 0.042)	19.72%
Married/cohabiting	−0.009 (−0.023, 0.006)	−12.68%
**SES**
Education (years)	0.009 (−0.024, 0.042)	12.68%
Income	0.033 (−0.018, 0.083)	46.48%
**Life styles**
Smoking	−0.001 (−0.007, 0.005)	−1.41%
Drinking	−0.001 (−0.009, 0.006)	−1.41%
Exercise	0.055^***^ (0.021, 0.089)	77.46%
**Health status**
ADL limitation	−0.037 (−0.090, 0.015)	−52.11%
Chronic disease	−0.003 (−0.011, 0.005)	−4.23%

95% confidence intervals (CI) in parentheses.

N, number; ADL limitation, activities of daily living limitation.

*** *p* < 0.001.

Table 7 shows the contribution of individual characteristics to all models explaining the gap in depressive symptoms. Columns 1–3 correspond to models 1–3 in Tables 4–6. Table 7 clearly shows that a large part of the gap in depressive symptoms in all the models is explained by years of education and income. The proportion of ADL limitation in the explanatory difference among older adults in different main residential locations was relatively extreme, accounting for only 2.28% of urban residents and rural–urban migrants. However, the proportion of ADL limitation between rural residents and rural–urban migrants was as high as −52.11%.

**Table 7:** Contribution of individual characteristics to the explained gap in depressive symptoms (weighted).

**Model**	**1**	**2**	**3**
Explained difference	499.75%	−43,100.00%	−17.49%
**Explanatory variables**
**Sociodemographic factors**
Age	0.50%	−1.47%	15.49%
Male	−0.25%	−0.15%	19.72%
Married/cohabiting	0.74%	1.39%	−12.68%
**SES**
Education (years)	38.53%	48.72%	12.68%
Income	49.90%	37.12%	46.48%
**Life styles**
Smoking	0.20%	1.78%	−1.41%
Drinking	0.60%	−3.25%	−1.41%
Exercise	8.64%	14.77%	77.46%
**Health status**
ADL limitation	2.28%	0.46%	−52.11%
Chronic disease	−1.14%	0.62%	−4.23%

Columns 1–3 correspond to models 1–3 in Tables 4–6.

ADL limitation, activities of daily living limitation.

## Discussion

Our research shows that the prevalence of depressive symptoms among older adults in different main residential locations differs significantly. The depressive symptom scores of the rural population living in cities were significantly higher than those of the urban and rural residents. This is consistent with previous studies on older adults (35, 36). The analysis between the different groups showed that education and income were the most significant explanatory factors. This finding is consistent with the results of previous studies (37, 38).

The results of previous studies are inconsistent with regard to the relationship between main residential locations and depressive symptoms. Regarding high-income countries, the prevalence of depressive symptoms in urban areas was higher in 2017 (39). Regarding low- and middle- income countries, however, especially China, the prevalence of depressive symptoms was higher in rural areas in 2011 (38–40). This phenomenon is attributable to the difference in the economic level between high-income countries and low- and middle-income countries, resulting in different environmental characteristics in urban and rural areas and differences in basic public services enjoyed by residents.

Some studies show that internal migration populations from rural-to-urban areas have a lower rate of depression in China (11, 41). However, our study shows that older adults who lived in rural and urban areas for a long time had lower depressive symptom scores than rural–urban migrants, which is inconsistent with the findings of previous studies. In the process of urbanization, the internal migration population must overcome the social, physical, and even psychological challenges driven by migration. After a long period of cultural adaptation, it is possible to successfully migrate. However, for immigrants who do not have sufficient time to adapt to the environment in which they move, their psychological status is worrying.

This study shows that older adults with low depressive symptom scores had longer years of education, better income, and more exercise. In urban and rural areas, with relatively basic leisure facilities, exercise is an effective way to relieve stress (42, 43). When rural–urban migrants move to a new environment, they have to work for a living and often ignore healthy behaviors, such as exercise. However, older adults who have lived in urban or rural areas for a long time are stable and have more time and experience to pay attention to their own health. The prevalence of depressive symptoms among urban residents was the lowest, and a large part of the explanation was related to the social environment in our study. A high-quality urban environment is conducive to the mental health of urban residents. The depressive symptom score of rural–urban migrants was the highest, which can be explained by the institutional inequality facing rural migrants in Chinese urban areas. Owing to the existence of the hukou system, rural–urban migrants entering urban areas and rural–urban migrants can only receive the most basic pensions, which are not enough to support urban life. In addition, the living pressure on children is relatively high, while older adults have to worry about pension issues. At this stage, the physical and mental health of rural–urban migrants is significantly challenged.

The ratios of ADL limitation were the opposite among older adults with different main residential locations. This phenomenon can be explained by the acculturation theory. Following the increase in the age of the rural–urban migrants, the loss of their physical health has become more serious, especially for rural–urban migrants with ADL limitation. They do not have access to urban hukou and cannot enjoy the preferential medical resources of the urban population, thereby increasing their psychological burden. Older adults with more years of education have struggled in the urban areas for decades. However, their urban identity cannot be recognized, resulting in excessive psychological pressure and an increasing in the possibility of suffering from depressive symptoms.

In summary, this study decomposes the gap in the depressive symptom score between different main residential locations, positing that a long-term and stable residential environment may reduce the risk of depression among older adults.

The study of reducing depressive symptom scores among older adults provides us with a lot of inspiration. First, due to the development of urbanization, a large number of rural people go to the city, resulting in an increase in urban pressure. It is a good measure to attract more people to the countryside through the revitalization of the countryside. Thus, providing more development opportunities to rural areas is key to narrowing the gap between urban and rural areas. By tilting more resources to rural areas, for example, to attract more young people to return to their hometowns for the benefit of developing industries in rural talents, more ambitious and capable people can go to rural areas and build beautiful villages. We can improve education, provide more job opportunities, help people establish a correct concept of health, develop healthy behavior, and obtain more social support (44). More importantly, it is crucial to set up psychological counseling stations in grassroots medical and health institutions and install sports building materials in communities and villagers' activity centers to further improve the mental health of rural people. As urbanization is an inevitable process of human progress, the coverage of the medical security system for the rural–urban migrants should be more comprehensive and consider the physical and mental health of older adults. The pension treatment standard should be gradually unified to achieve consistency between urban and rural areas.

This study shows that education and income are the most important explanatory factors for the impact of main residential location on depressive symptoms, which is not only applicable to China but also to other low- and middle-income countries. Education and income, as symbols of socioeconomic status, not only affect the physical health of older adults but also have a non-negligible effect on the mental health of the population. Among older adults in low- and middle-income countries, those with a long education are more able to take on light jobs and increase their income. This means that they do not need to worry about food or clothing in their later lives. By contrast, those with a short education are relatively more likely to work hard. Therefore, improving people's education is a priority in low- and middle-income countries.

## Strengths and limitations

The main advantages are as follows: First, we used the CLHLS, a representative sample of long-lived older adults in China, to examine the mental health of long-lived older adults and add some health-related variables, such as ADL limitation and chronic diseases. Second, we used the Blinder–Oaxaca decomposition method to quantify the explainable factors that cause the gap in the depressive symptoms among older adults in urban and rural areas and to clarify the share of each factor in the effect of depressive symptoms.

Our study has several limitations. First, it is a cross-sectional study, which does not support the causal inference between main residential locations and depressive symptoms among older adults. It may be difficult to infer why the gap in the depressive symptoms among older adults under different main residential locations cannot be explained. Second, we may not be able to explain some of the factors that influence the difference between the groups in the unexplained part of the depressive symptom gap (45). Third, using the CES-D scale to evaluate depressive symptoms can only provide the prevalence rate of depressive symptoms but cannot provide the prevalence rate of depression.

## Conclusion

This study reports that the scores of depressive symptoms among older adults differed according to main residential locations. The depressive symptom score of older adults living permanently in urban or rural areas was lower than that of rural–urban migrants. Women living alone, with lower education, lower income, no drinking, no exercise, ADL restrictions, and chronic diseases were associated with higher depressive symptoms among older adults. The most important reasons for the urban–rural gap in depressive symptoms are education and income.

The results of this study have several practical implications. The results showed that healthcare policymakers and professionals need to consider a number of factors when considering higher depressive symptom scores. These factors include the demographic characteristics, socioeconomic status, and behavioral lifestyle of different countries. Health education and health promotion strategies should be used to improve the health literacy of older adults and prevent an increase in their depressive symptom scores. Particularly, primary health institutions should focus on the mental health of older adults and provide mental health education and psychological counseling. Corresponding policies should be formulated according to the characteristics of the country. In this study, there was a significant difference in the depressive symptom scores between rural–urban migrants and permanent residents. The development of industries in rural areas attracts more young people to return to their hometowns for benefits. It is necessary for national health insurance policies to pay more attention to temporary residents and to unify urban and rural pension standards. There is a need for further research to explore why different factors have different effects on depressive symptoms among older adults.

## Data availability statement

Publicly available datasets were analyzed in this study. This data can be found at: http://opendata.pku.edu.cn/dataverse/CHADS.

## Ethics statement

The studies involving human participants were reviewed and approved by the Ethical Review Committee of Peking University (IRB00001052-13074). The patients/participants provided their written informed consent to participate in this study. Written informed consent was obtained from the individual(s) for the publication of any potentially identifiable images or data included in this article.

## Author contributions

YC and QY designed the study and responsible for the collation and analysis of the data. YC analyzed the data and wrote the first draft of the manuscript. QY supported the interpretation of the data. WL contributed to formal analysis. GZ contributed to conceptualization, methodology, and editing. All authors contributed to the research programme, contributed to the final manuscript, and approved the final manuscript version.

## Funding

This study was supported by the National Natural Science Foundation of China (grant No. 71774102).

## Conflict of interest

The authors declare that the research was conducted in the absence of any commercial or financial relationships that could be construed as a potential conflict of interest.

## Publisher's note

All claims expressed in this article are solely those of the authors and do not necessarily represent those of their affiliated organizations, or those of the publisher, the editors and the reviewers. Any product that may be evaluated in this article, or claim that may be made by its manufacturer, is not guaranteed or endorsed by the publisher.

## Abbreviations

CLHLS: Chinese Longitudinal Healthy Longevity Survey
CES-D: Center for Epidemiologic Studies Short Depression Scale
SES: socioeconomic status
ADL limitation: activities of daily living limitation
SD: standard deviation.
